# Gastric Inflation in Prehospital Cardiopulmonary Resuscitation: Aspiration Pneumonia and Resuscitation Outcomes

**DOI:** 10.31083/j.rcm2407198

**Published:** 2023-07-12

**Authors:** Tae Youn Kim, Soyeong Kim, Sang Il Han, Sung Oh Hwang, Woo Jin Jung, Young Il Roh, Kyoung-Chul Cha

**Affiliations:** ^1^Department of Emergency Medicine, Dongguk University Ilsan Hospital, Dongguk University College of Medicine, 10326 Goyang, Republic of Korea; ^2^Department of Emergency Medicine, Yonsei University Wonju College of Medicine, 26426 Wonju, Republic of Korea

**Keywords:** airway management, cardiopulmonary resuscitation, gastric inflation, prehospital emergency care, ventilation

## Abstract

**Background:**

Gastric inflation (GI) can induce gastric regurgitation and subsequent aspiration pneumonia, which can prolong intensive care unit stay. However, it has not been verified in patients with out-of-hospital cardiac arrest (OHCA). This study aimed to investigate the incidence of GI during prehospital resuscitation and its effect on aspiration pneumonia and resuscitation outcomes in patients with out-of-hospital cardiac arrest.

**Methods:**

This was a multicenter, retrospective, observational study. Patients with non-traumatic OHCA aged >19 years who had been admitted to the emergency department were enrolled. Patients who received mouth-to-mouth ventilation during bystander cardiopulmonary resuscitation (CPR) were excluded from the evaluation owing to the possibility of GI following bystander CPR. Patients who experienced cardiac arrest during transportation to the hospital who were treated by the emergency medical service (EMS) personnel, and those with a nasogastric tube at the time of chest or abdominal radiography were also excluded. Radiologists independently reviewed plain chest or abdominal radiographs immediately after resuscitation to identify GI. Chest computed tomography performed within 24 h after return of spontaneous circulation was also reviewed to identify aspiration pneumonia.

**Results:**

Of 499 patients included in our analysis, GI occurred in approximately 57% during the prehospital resuscitation process, and its frequency was higher in a bag-valve mask ventilation group (n = 70, 69.3%) than in the chest compression-only cardiopulmonary resuscitation (n = 31, 55.4%), supraglottic airway (n = 180, 53.9%), and endotracheal intubation groups (n = 3, 37.5%) (*p* = 0.031). GI was inversely associated with initial shockable rhythm (adjusted odds ratio [OR] 0.53; 95% confidence interval [CI]: 0.30–0.94). Aspiration pneumonia was not associated with GI. Survival to hospital discharge and favorable neurologic outcomes were not associated with GI during prehospital resuscitation.

**Conclusions:**

GI in patients with OHCA was not associated with the use of different airway management techniques.

## 1. Introduction

Approximately 360,000 patients experience out-of-hospital cardiac arrest (OHCA) annually in the United States of America, but the survival rate remains low [1]. To ensure survival after cardiac arrest, high-quality cardiopulmonary resuscitation (CPR) has been emphasized in CPR guidelines [2]. Advanced airway management, which promotes continuous chest compression during CPR, is one of the key elements for ensuring high-quality CPR, so most rescuers try to secure an advanced airway during resuscitation [3]. However, because similar resuscitation outcomes between basic, and advanced airway management have been reported in previous studies, either basic or advanced airway management in OHCA has been recommended in recent CPR guidelines. Although still controversial, the CPR guidelines consider endotracheal intubation (ETI) to be a definitive airway management technique during resuscitation to provide optimal ventilation and minimize the risk of aspiration. However, ETI is highly dependent on the practitioner’s skill, making it difficult to make global recommendations [2, 4, 5]. However, it is difficult to perform ETI during CPR, even for experienced physicians, so a supraglottic airway (SGA) was introduced as an alternative [6, 7]. Because it is rapid and simple, and requires less training, many emergency medical service (EMS) personnel choose the SGA for primary advanced airways [8]. As no difference in resuscitation outcomes between these airway management devices has been reported, healthcare providers have tended to select the airway devices based on their own familiarity during CPR [9].

However, the risks of gastric inflation (GI) induced using bag-mask ventilation and incomplete oropharyngeal- or tracheal-securing systems using SGA should be considered because GI can cause aspiration pneumonia, which is a risk factor for prolonged mechanical ventilation duration and length of stay in the intensive care unit in patients post-cardiac arrest [4, 10, 11].

This study aimed to evaluate the effects of prehospital ventilation on GI incidence and resuscitation outcomes in patients with OHCA.

## 2. Methods

### 2.1 Study Design and Setting 

This multicenter, retrospective, observational study involved two university hospitals. Annually, approximately 43,000 and 40,000 patients visit each hospital’s emergency department (ED), including approximately 120 and 60 patients with OHCA, respectively. This study was approved by the Institutional Review Board (IRB) of Wonju Severance Christian Hospital (IRB No. CR320049) and the IRB of Dongguk University Ilsan Hospital, Dongguk University (IRB No. DUIH 2021-11-010) and informed consent was waived because of the retrospective nature of the study and anonymous clinical data used for analysis.

In South Korea, EDs are designated as levels 1–3; levels 1 (38 facilities) and 2 (119 facilities) have the highest volumes, with emergency physicians staffed at all times, and level 3 (261 facilities) can be staffed by general physicians [12]. Wonju Severance Christian Hospital is a level 1 ED, and Dongguk University Ilsan Hospital is a level 2 ED.

Patients who experience OHCA are managed by three emergency medical technicians (EMTs) who are dispatched from a fire department. The EMTs provide both basic and advanced life support, including defibrillation, intravenous access, epinephrine administration, and advanced airway management, for a minimum of 5 min at the scene under the medical direction of a physician. EMTs perform CPR with chest compression only or as a 30:2 compression-to-ventilation ratio if the advanced airway is not secured, and asynchronous ventilation is delivered every 6 s once the advanced airway has been secured. If return of spontaneous circulation (ROSC) cannot be achieved, such patients are transported to the nearest ED while EMTs continue to perform CPR in the ambulance. Once a patient with OHCA arrives at the ED, the patient is transferred to the resuscitation unit immediately, while the EMT continues CPR. After arrival at the resuscitation unit, another healthcare provider takes over chest compressions, and an emergency physician performs ETI immediately without bag-mask ventilation. Other advanced life support is performed according to the current Korean advanced life support guidelines [13]. Under direct medical supervision, EMTs can provide clinical care to patients with OHCA, including CPR, advanced airway management, and administering intravenous fluids and epinephrine injections. EMTs in Korea are classified as level-1 or level-2 according to their work scope and qualifications. Level 1 EMT qualifications include graduation from a paramedic school (3- to 4-year curriculum) at a university or community college. It also requires at least 2 years of clinical experience, and there are many level 1 paramedics, including nurses, working in out-of-hospital settings. Paramedic schools have six courses, 147 h of training, and a specific curriculum for advanced airway management. Level 1 EMTs mainly perform advanced airway management in patients with OHCA. Essential education for EMTs in Korea consists of 4-h theoretical classes and practical classes using mannequins, with flexible education conducted every year [12, 14, 15].

Portable chest and abdominal radiographs are obtained immediately after resuscitation to differentiate the potential cause of cardiac arrest irrespective of survival.

### 2.2 Patients

Patients with non-traumatic OHCA aged >19 years who had been admitted to the ED and who had undergone chest or abdominal radiography from December 2015 to December 2020 were enrolled in this study. A cutoff age of 19 years was chosen because the EMTs used only i-gel sizes 3 or 4 in this setting; thus, we limited the study to adults because i-gel application may be inappropriate in relatively small children and this may bias the incidence of gastric inflation. Patients who received mouth-to-mouth ventilation during bystander CPR were excluded from the evaluation due to the possibility of GI following bystander CPR. Patients who had a cardiac arrest during transportation to hospital who received treatment by EMS personnel and patients with nasogastric tube at the time acquired chest or abdominal radiography were also excluded.

### 2.3 Study Variables

**Fig. 1.**
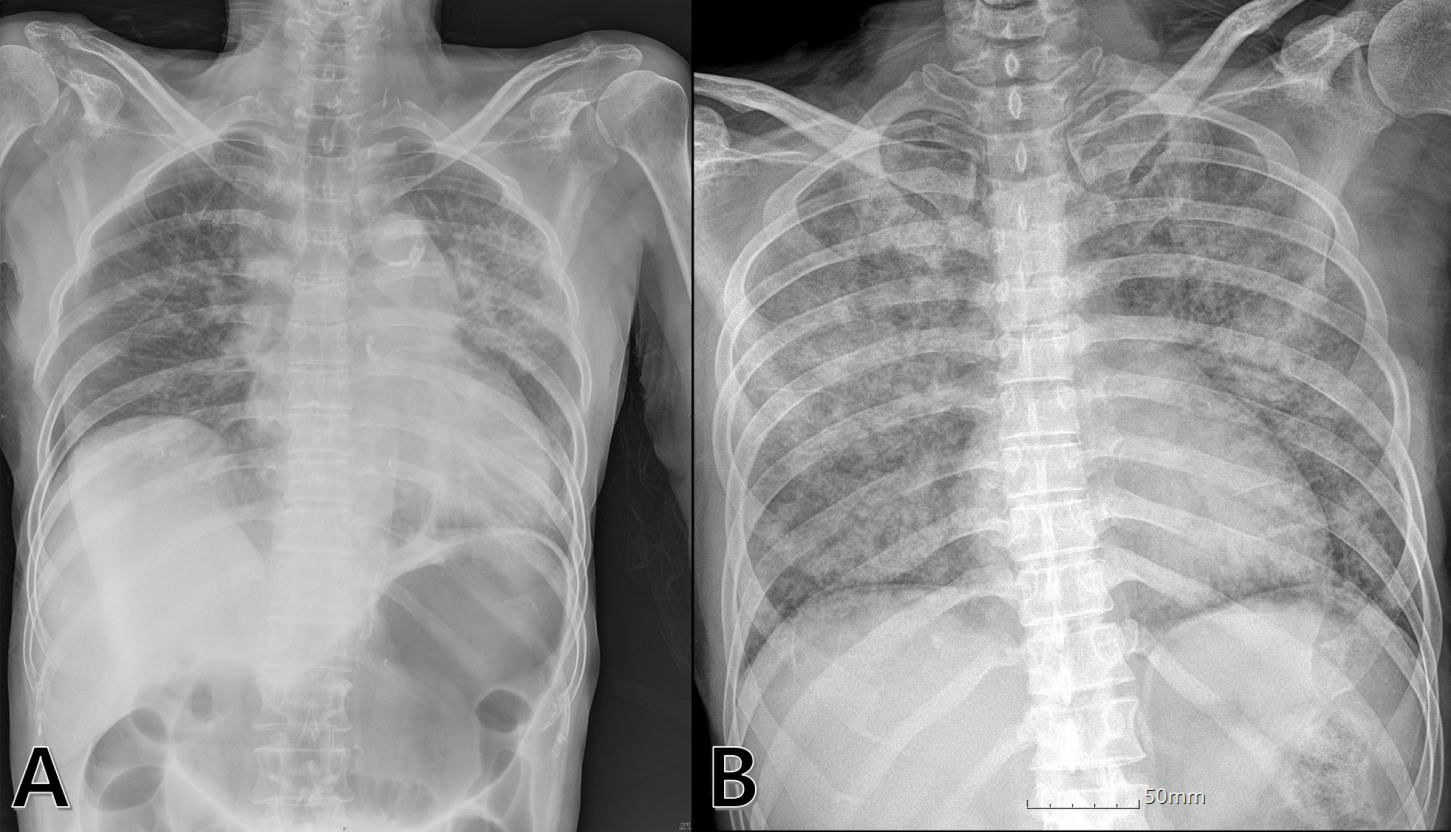
**An example of a plain chest radiograph with (A) and without (B) gastric inflation after prehospital resuscitation**.

The following clinical and laboratory parameters were obtained from the medical records: age, sex, witness of cardiac arrest, bystander CPR, estimated total cardiac arrest time, initial presenting rhythm, total duration of CPR, total dose of epinephrine administered, cumulative defibrillation energy, presence of aspiration pneumonia, survival to hospital discharge, and neurologic outcome at hospital discharge. Chest and abdomen radiographs were taken in the emergency department within 1 hour. GI was defined as a massively distended stomach on chest and abdominal radiography [10] (Fig. 1). Aspiration pneumonia was defined as bilateral perihilar, ill-defined, alveolar consolidations, multifocal patchy infiltrates, and/or segmental or lobar consolidation on chest computed tomography (CT) acquired within 24 h after ROSC [16, 17]. Two radiologists unrelated to our study reviewed the plain chest and abdomen radiographs and chest CT scans and confirmed GI or aspiration pneumonia. If there was a disagreement concerning a radiologic reading, the two radiologists discussed and confirmed the findings together. The EMS response time was defined as the time interval between the call for EMS and EMS arrival at the scene. The scene time interval was defined as the duration in which EMS personnel provided basic and advanced life support at the scene. Transport time was defined as the time interval between EMS departure from the scene and arrival at the ED in each hospital. A favorable neurological outcome was defined as a cerebral performance category (CPC) score of 1 or 2.

### 2.4 Statistical Analysis

Continuous data are presented as means with standard deviations or medians (interquartile ranges), according to the normality test. Normally distributed data were assessed using a Shapiro–Wilk test. Categorical variables are presented as counts and percentages. Continuous data were analyzed using Student’s *t*- or Mann–Whitney *U* tests, as appropriate. Categorical data were analyzed using the chi-square or Fisher’s exact test, as appropriate. The interclass correlation coefficient (Cronbach’s alpha) was calculated to evaluate interobserver reliability for presenting GI on chest or abdominal radiography. To evaluate the factors contributing to clinical outcomes, including development of GI, aspiration pneumonia, survival to discharge, and favorable neurologic outcome, univariable and multivariable logistic regression analyses were performed, which are presented with odds ratios (OR) and 95% confidence intervals (CI). After verifying the log-linearity of the continuous variables by Box–Tidwell transformations and testing the goodness of fit of the regression model with the Hosmer–Lemeshow goodness-of-fit test, a logistic regression analysis was performed. Variables with a *p*-value < 0.2 in univariable logistic regression analysis were included in the multivariable logistic regression analysis. Two-sided *p*-value of <0.05 were considered statistically significant. To calculate the effect size, Cramer’s V and Hedges’ G coefficients were calculated. All analyses were performed using the SPSS ver. 25 (IBM Corp., Armonk, NY, USA).

## 3. Results

### 3.1 General Characteristics

**Fig. 2.**
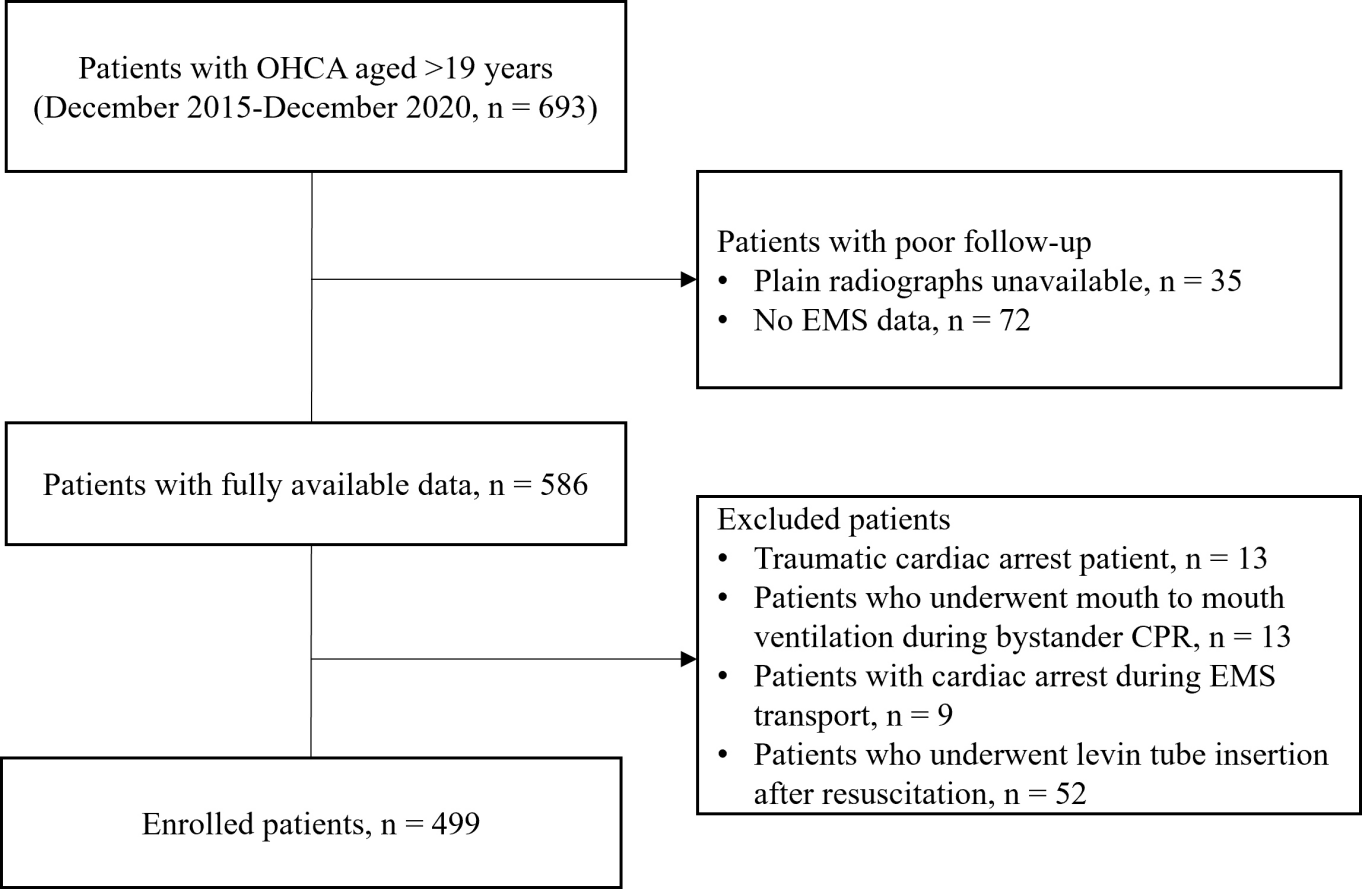
**Flowchart of patient screening and selection during the study enrollment process**. Abbreviations: CPR, cardiopulmonary resuscitation; EMS, emergency medical service; OHCA, out-of-hospital cardiac arrest.

During the study period, 693 adult patients with OHCA had been admitted to the ED. Among them, we excluded patients by exclusion criteria. In total, 499 patients were enrolled in the final analysis (Fig. 2).

GI was more frequently observed with bag-valve mask ventilation than with SGA or ETI (*p *= 0.031). GI was more frequently observed with female (*p *= 0.030) Initial shockable rhythm was more frequently observed in patients without GI (no GI [NGI] group, *p *= 0.015). The other variables did not differ between the groups (Table 1). The effect size coefficients calculated are shown in the **Supplementary Table**. Interobserver reliability values for diagnosing GI and aspiration pneumonia were excellent (Cronbach alpha, 0.87 and 0.9, respectively).

**Table 1.** **Baseline characteristics of patients**.

Variables	NGI group (n = 215)	GI group (n = 284)	*p*-value
Age (years)	69.5 ± 16.4	71.6 ± 14.5	0.24
Male, n (%)	139 (64.7%)	155 (54.6%)	0.030
Witness of cardiac arrest	135 (62.8%)	166 (58.5%)	0.37
Airway management			0.031
Compression only	25 (11.6%)	31 (10.9%)	
Bag-valve mask ventilation	31 (14.4%)	70 (24.6%)	
Supraglottic airway	154 (71.6%)	180 (63.4%)	
Endotracheal intubation	5 (2.3%)	3 (1.1%)	
Bystander CPR	99 (46.0%)	148 (52.1%)	0.21
Initial shockable rhythm	39 (18.1%)	29 (10.2%)	0.015
Cumulative defibrillation energy (J)	0 (0–2400)	0 (0–7600)	0.051
Total collapse time (min)	31 (2–157)	30 (12–255)	0.46
Total duration of CPR (min)	23 (1–130)	22 (3–240)	0.21
EMS response time (min)	8 (1–55)	8 (1–52)	0.32
Scene time interval (min)	9 (0–127)	9 (0–65)	0.97
Transport time (min)	11 (1–77)	11 (1–175)	0.51
Total administered dose of epinephrine (mg)	5 (0–16)	5 (0–33)	0.48

Variables are presented as mean ± standard deviation or median (interquartile range). Abbreviations: CPR, cardiopulmonary resuscitation; EMS, emergency medical service; GI, patients with gastric inflation; NGI, patients without gastric inflation.

### 3.2 Comparison of Clinical Outcomes According to Airway Management

The clinical outcomes according to airway management are presented in Table 2. GI was most frequently observed in the bag-valve mask ventilation group compared to the other techniques. (*p* = 0.031). The frequency of aspiration pneumonia did not differ between the groups (*p = *0.082) (Table 2).

**Table 2.** **Comparison of clinical outcomes of patients according to airway management**.

	Chest compression only (n = 56)	Bag-valve mask group (n = 101)	SGA group (n = 334)	ETI group (n = 8)	*p*-value
GI	31 (55.4%)	70 (69.3%)	180 (53.9%)	3 (37.5%)	0.031
ROSC	20 (35.7%)	31 (30.7%)	109 (32.6%)	2 (25.0%)	0.89
Aspiration pneumonia	6 (10.7%)	11 (10.9%)	60 (18.0%)	1 (12.5%)	0.082
Survival discharge	13 (23.2%)	22 (21.8%)	50 (15.0%)	2 (25.0%)	0.22
CPC 1–2	5 (8.9%)	9 (8.9%)	17 (5.1%)	1 (12.5%)	0.37

Abbreviations: CPC, cerebral performance category; ETI, endotracheal intubation; GI, gastric inflation; ROSC, return of spontaneous circulation; SGA, supraglottic airway.

### 3.3 Factors Associated with Gastric Inflation during Prehospital Resuscitation

Male sex was associated with less development of GI in the univariate analysis, but no association was found in the multivariable logistic regression analysis. Initial shockable rhythm was associated with less development of GI in univariate and multivariable logistic regression analyses (Table 3).

**Table 3.** **Univariable and multivariable logistic regression analyses for verifying factors related to GI**.

Variable	Univariable logistic regression		Multivariable logistic regression	
	Crude OR	95% CI	Adjusted OR	95% CI
Age	1.00	0.99–1.00	1.00	0.99–1.00
Male	0.65	0.45–0.94	0.71	0.48–1.04
Witness	0.83	0.58–1.19		
Airway				
Compression-only	(reference)		(reference)	
Bag-valve mask	1.82	0.92–3.57	1.90	0.96–3.78
SGA	0.94	0.53–1.66	0.93	0.52–1.66
ETI intubation	0.48	0.10–2.22	0.48	0.10–2.30
Bystander CPR	1.27	0.89–1.81	1.37	0.95–1.99
Initial shockable rhythm	0.51	0.30–0.86	0.53	0.30–0.94
Total cardiac arrest time (min)	1.00	0.99–1.00		
Total duration of CPR (min)	0.99	0.99–1.00		
EMS response time (min)	1.00	0.99–1.01		
Scene time interval (min)	0.99	0.99–1.00		
Transport time (min)	1.00	0.99–1.01		

Abbreviations: CI, confidence interval; CPR, cardiopulmonary resuscitation; EMS, emergency medical service; ETI, endotracheal intubation; GI, gastric inflation; OR, odds ratio; SGA, supraglottic airway.

### 3.4 Factors Associated with the Development of Aspiration Pneumonia in Resuscitated Patients

Initial shockable rhythm was inversely associated with the development of aspiration pneumonia (Table 4).

**Table 4.** **Univariable and multivariable logistic regression analyses to verify factors related to aspiration pneumonia**.

Variable	Univariable logistic regression		Multivariable logistic regression	
	Crude OR	95% CI	Adjusted OR	95% CI
Age	1.00	1.00–1.01	1.00	1.00–1.00
Male	0.71	0.38–1.34		
Witness	0.90	0.43–1.87		
Airway				
Chest compression-only	(reference)			
Bag-valve mask	1.28	0.38–4.28	1.18	0.31–4.49
SGA	2.85	1.02–7.98	2.27	0.71–7.26
ETI	2.33	0.12–43.79	6.25	0.24–158.68
Bystander CPR	0.87	0.47–1.63		
GI	1.47	0.78–2.77		
Initial shockable rhythm	0.18	0.07–0.45	0.25	0.08–0.71
Total cardiac arrest time (min)	1.00	0.99–1.00		
Total duration of CPR (min)	1.00	0.99–1.00		
EMS response time (min)	1.01	0.99–1.03	1.01	0.99–1.03
Scene time interval (min)	1.00	0.99–1.01	1.00	0.99–1.01
Transport time (min)	0.99	0.98–1.00	0.99	0.98–1.00

Abbreviations: CI, confidence interval; CPR, cardiopulmonary resuscitation; EMS, emergency medical service; ETI, endotracheal intubation; GI, gastric inflation; OR, odds ratio; SGA, supraglottic airway.

### 3.5 Factors Associated with Resuscitation Outcomes

There was no statistical association between resuscitation outcomes and airway management in multivariable regression analyses. The adjusted OR of gastric inflation was also not statistically significant with resuscitation outcomes (Table 5).

**Table 5.** **Multivariable logistic regression analysis to verify factors related to ROSC, survival to hospital discharge, and favorable neurologic outcome**.

Variable	Return of spontaneous circulation adjusted ${\mathrm{OR}}^{a}$		Survival hospital discharge adjusted ${\mathrm{OR}}^{a}$		Favorable neurologic outcome adjusted ${\mathrm{OR}}^{a}$	
	OR	95% CI	OR	95% CI	OR	95% CI
Airway						
Compression-only	(reference)		(reference)		(reference)	
Bag-valve mask	0.69	0.31–1.37	0.77	0.33–1.80	0.75	0.18–3.13
SGA	0.85	0.45–1.60	0.58	0.27–1.24	0.40	0.11–1.49
ETI	0.53	0.09–3.15	1.08	0.15–7.64	1.33	0.06–29.96
GI	1.32	0.87–2.00	1.52	0.89–2.58	1.90	0.75–4.80

^a^Controlling for centered age, sex, witness, bystander CPR, and initial shockable rhythm. Abbreviations: CI, confidence interval; CPR, cardiopulmonary resuscitation; ETI, endotracheal intubation; GI, gastric inflation; OR, odds ratio; ROSC, return of spontaneous circulation; SGA, supraglottic airway.

## 4. Discussion

In this study, we observed that GI occurred in approximately 57% of patients during the prehospital resuscitation process, but that resuscitation outcomes (ROSC, survival to discharge, and favorable neurological outcome) were not associated with GI, and they also had no effect on the development of aspiration pneumonia or resuscitation outcomes. The modality of airway management was also not associated with GI. Therefore, it is desirable to follow the current CPR guidelines that recommend using either basic or advanced airways during CPR [2, 5].

It has previously been well established that GI causes aspiration of gastric contents, which increases the risk of developing aspiration pneumonitis and that GI is a risk factor for a poor outcome in patients post-cardiac arrest [11, 18]. In addition, GI can result in an increase in intra-abdominal pressure, which can reduce venous return and increase afterload, resulting in an increase in systemic vascular resistance. GI can also lead to a decrease in functional residual capacity, which may have further implications on ventilation [19, 20]. Therefore, it is necessary to remain vigilant concerning the risk of GI during and after resuscitation to reduce avoidable complications. Recent CPR guidelines recommend that healthcare providers use either basic or advanced airway management during CPR, because they show similar effects on resuscitation outcomes [2, 5]. Although ETI is the best method in which to secure the airway during resuscitation, SGA has been introduced as an alternative to ETI during resuscitation because it has the advantages of a high success rate, low complications, and ease of learning compared with ETI [21]. The SGAs also have the advantage of fast insertion times, which is beneficial for a high chest compression fraction (CCF) [22]. In particular, i-gel, a type of SGA, has a soft material and non-inflatable cuff designed to create an anatomical seal around the pharyngeal and laryngeal cavities; therefore, patients experience fewer sore throats and less oral or pharyngeal damage due to SGA insertion, which is the most common type of advanced airway device for patients with OHCA in the EMS system in the Republic of Korea [23, 24, 25]. However, i-gel can induce GI more frequently than other SGA types because it has been shown to lower oropharyngeal leak pressure compared with other SGAs [26]. This may explain why the frequency of GI was found to be high in this study, as approximately 53% of patients with cardiac arrest received advanced airway management using i-gel. GI may occur if i-gel is not fixed in the correct position. To prevent position change after insertion, it is recommended to fix i-gel accurately using the fixation strap enclosed in the i-gel kit. However, the i-gel kit distributed to EMSs in the Republic of Korea does not include a fixation strap so EMTs often fix it with tape according to their assessment after i-gel insertion. An incomplete seal can induce air leakage during artificial ventilation and inflation. One study showed that position change was greatest when i-gel was not fixed, whereas position change occurred least when fixing with the i-gel fixation strap compared with using Durapore tape, Multipore tape, or other fixation straps [27]. Therefore, healthcare providers should use SGAs with accurate positioning and fixation to minimize air leakage and GI.

GI was not associated with aspiration pneumonia in this study. One previous study reported that GI might contribute to 12% of gastric regurgitation in patients with cardiac arrest on bag-valve mask ventilation [28]. As reported previously, the association between GI and aspiration pneumonia might be due to a discrepancy between GI and corresponding gastric regurgitation. Various pathogens and varying incubation periods of aspiration pneumonia might also be reasons for the discrepancy between gastric regurgitation and aspiration pneumonia observed on the chest CT scans because we evaluated all chest CT scans within 24 h after ROSC [29]. Nasogastric tube insertion in all patients who received post-cardiac arrest care might be also one of the reasons that there is no association between GI and aspiration pneumonia. Further prospective observational studies with serial evaluations of chest CT scans are needed to verify a more accurate relationship between GI and aspiration pneumonia.

In this study, GI was more frequently observed in patients with non-shockable rhythm A lower esophageal sphincter pressure decreased rapidly during circulatory collapse, which might be one of the reasons for GI in patients with cardiac arrest [30]. However, the distal esophagus could be cramped after defibrillation in patients with shockable rhythm, which might be a reason why GI was infrequently observed in patients with shockable rhythm [31]. Because acute respiratory failure and metabolic acidosis are the most common causes of cardiac arrest with non-shockable rhythm, compensatory hyperventilation and corresponding aerophagia can continue until the development of cardiac arrest [32, 33], which might be another reason for the high frequency of GI in patients with non-shockable rhythm.

This study has several limitations. First, it was a retrospective study based on medical records; therefore, completely controlling for confounders was challenging. Second, there was a possibility of selection bias because aspiration pneumonia was diagnosed only in patients with ROSC and an acquired chest CT. Third, the effect of mouth-to-mouth ventilation on GI was not evaluated because the medical records contained very little information concerning bystander ventilation. Fourth, EMTs were most likely to use i-gel sizes 3 or 4. This may have biased the occurrence of gastric inflation because i-gel application may be inappropriate depending on the patient’s size. Fifth, GI was diagnosed based on image reading, although this does not represent an objective definition. Sixth, there was a large imbalance in the ETI group (n = 8), which may be a limitation that should be considered cautiously when interpreting some of the results. Finally, it is possible that the presence of GI was affected due to the volume and frequency of artificial ventilation, even though all EMTs performed basic and advanced life support according to current CPR guidelines and some patients may have received multiple airway management techniques during resuscitation, and this could potentially lead to misclassification bias.

## 5. Conclusions

GI in patients with OHCA was not associated with the use of different airway management techniques. Prospective observational study might be needed to verify the effect of GI on aspiration pneumonia or resuscitation outcomes more precisely.

## Data Availability

Data cannot be shared publicly because of consent of personal information. The data can be accessed under the permission from corresponding author. The contact information is as follows: chaemp@yonsei.ac.kr.

## Abbreviations

GI, gastric inflation; OHCA, out-of-hospital cardiac arrest; CPR, cardiopulmonary resuscitation; ETI, endotracheal intubation; SGA, supraglottic airway; EMT, emergency medical technicians; ED, emergency department; ROSC, return of spontaneous circulation; CT, computed tomography.

## Supplementary Material

Supplementary material associated with this article can be found, in the online version, at https://doi.org/10.31083/j.rcm2407198.
