# Supplementary material for: Gastric Inflation in Prehospital Cardiopulmonary Resuscitation: Aspiration Pneumonia and Resuscitation Outcomes
Source: Rev Cardiovasc Med. 2023 Jul 12;24(7):198. doi: 10.31083/j.rcm2407198 (PMC11266457; doi:10.31083/j.rcm2407198)
Supplement: Supplementary file 1 [file 2153-8174-24-7-198-s1.zip › Supplement table.pdf]

Supplementary table. *Effect size coefficients by gastric inflation*

Variables	Effect size
Age (years) [a]	0.137
Male sex, n (%)[b]	0.024
Witness of cardiac arrest [b]	0.326
Airway management	0.031
Compression only [b]	-
Bag-valve mask ventilation [b]	-
Supraglottic airway [b]	-
Endotracheal intubation [b]	-
Bystander CPR [b]	0.180
Initial shockable rhythm [b]	0.011
Total collapse time (min) [a]	0.065
Total duration of CPR (min) [a]	0.034
EMS response time (min) [a]	0.065
Scene time interval (min) [a]	0.045
Transport time (min) [a]	0.042
Total administered dose of epinephrine (mg) [a]	0.005

[a]Hedges G

[b]Cramer's V
