# Supplementary material for: Gastric Inflation in Prehospital Cardiopulmonary Resuscitation: Aspiration Pneumonia and Resuscitation Outcomes
Source: Rev Cardiovasc Med. 2023 Jul 12;24(7):198. doi: 10.31083/j.rcm2407198 (PMC11266457; doi:10.31083/j.rcm2407198)
Supplement: Supplementary file 1 [file 2153-8174-24-7-198-s1.zip › supplement table_RCM.docx]

***Supplementary table.*** *Effect size coefficients by gastric inflation*

| *Variables* | *Effect size* |
| --- | --- |
| *Age (years) ^a^* | *0.137* |
| *Male sex, n (%)^b^* | *0.024* |
| *Witness of cardiac arrest ^b^* | *0.326* |
| *Airway management* | *0.031* |
| *Compression only ^b^* | *-* |
| *Bag-valve mask ventilation ^b^* | *-* |
| *Supraglottic airway ^b^* | *-* |
| *Endotracheal intubation ^b^* | *-* |
| *Bystander CPR ^b^* | *0.180* |
| *Initial shockable rhythm ^b^* | *0.011* |
| *Total collapse time (min) ^a^* | *0.065* |
| *Total duration of CPR (min) ^a^* | *0.034* |
| *EMS response time (min) ^a^* | *0.065* |
| *Scene time interval (min) ^a^* | *0.045* |
| *Transport time (min) ^a^* | *0.042* |
| *Total administered dose of epinephrine (mg) ^a^* | *0.005* |

*^a^Hedges G*

*^b^Cramer’s V*
